# Development of Flexible Robot Skin for Safe and Natural Human–Robot Collaboration

**DOI:** 10.3390/mi9110576

**Published:** 2018-11-05

**Authors:** Gaoyang Pang, Jia Deng, Fangjinhua Wang, Junhui Zhang, Zhibo Pang, Geng Yang

**Affiliations:** 1 State Key Laboratory of Fluid Power and Mechatronic Systems, School of Mechanical Engineering, Zhejiang University, Hangzhou 310027, China; 21725090@zju.edu.cn (G.P.); 21725067@zju.edu.cn (J.D.); benzjh@zju.edu.cn (J.Z.); 2 Department of Mechanical and Process Engineering, Swiss Federal Institute of Technology Zurich, 8092 Zurich, Switzerland; fawang@student.ethz.ch; 3 ABB Corporate Research Sweden, 72178 Vasteras, Sweden; Pang.zhibo@se.abb.com

**Keywords:** flexible robot skin, human–robot collaboration, inkjet printing, heterogeneous system

## Abstract

For industrial manufacturing, industrial robots are required to work together with human counterparts on certain special occasions, where human workers share their skills with robots. Intuitive human–robot interaction brings increasing safety challenges, which can be properly addressed by using sensor-based active control technology. In this article, we designed and fabricated a three-dimensional flexible robot skin made by the piezoresistive nanocomposite based on the need for enhancement of the security performance of the collaborative robot. The robot skin endowed the YuMi robot with a tactile perception like human skin. The developed sensing unit in the robot skin showed the one-to-one correspondence between force input and resistance output (percentage change in impedance) in the range of 0–6.5 N. Furthermore, the calibration result indicated that the developed sensing unit is capable of offering a maximum force sensitivity (percentage change in impedance per Newton force) of 18.83% N^−1^ when loaded with an external force of 6.5 N. The fabricated sensing unit showed good reproducibility after loading with cyclic force (0–5.5 N) under a frequency of 0.65 Hz for 3500 cycles. In addition, to suppress the bypass crosstalk in robot skin, we designed a readout circuit for sampling tactile data. Moreover, experiments were conducted to estimate the contact/collision force between the object and the robot in a real-time manner. The experiment results showed that the implemented robot skin can provide an efficient approach for natural and secure human–robot interaction.

## 1. Introduction

Along with the paradigm shift from Industry 3.0 to Industry 4.0, industrial manufacturing is going through a process of changing toward flexible and intelligent manufacturing. Collaborative robots play an important role in smart factories since it contributes to the achievement of higher productivity and greater efficiency [1]. These changes are reflected in safety standards (the international standard ISO 10218 and the Technical Specification ISO/TS 15066: 2016) related to collaborative robotics and have led to the promising research and development to prevent human–robot collision and minimize the potential risks of hurting people [2,3]. In order to ensure robots to work with human beings with safety and effectiveness, there are critical issues that need to be addressed. For instance, the issue of the inherent safety of robots needs to be addressed by developing natural interfacing between the environment, humans, and robot peers [4]. However, due to the security issues that were not well resolved, a severe accident occurred on 1 July 2015: a 21-year-old technician was killed by a robot accidentally at the Volkswagen Kassel facility [5]. Although the advanced robot technology has been rapidly developed and applied, much attention has not been paid to the strategy for the intrinsic security of human–robot interaction [6,7]. For example, ABB (Zurich, Switzerland), a famous robot manufacturer, has recently launched a collaborative dual-arm robot, YuMi, which is embedded in the torque sensing unit to strengthen the robot security performance during human–robot collaboration [8]. This evolution has removed the safety barrier in the workspace between robot and human. However, the torque sensor is the only source for the YuMi robot to perceive stimulus from external environment. Due to the limited sensors, it’s difficult to guarantee that YuMi robots will completely avoid collision while assisting operation. Moreover, the rigid contact with severe impact between robot and human cannot satisfy the security requirements of collaborative robots [1]. Hence, the achievement of inherently safe and natural human–robot interaction is still a challenge in smart factories. Industrial manufacturing with the lack of key technology in safe and natural human–robot collaboration will lead to severe accidents.

Advances have been made in manufacturing of flexible electronics and functional materials [9]. These advanced technology endow the soft sensor with excellent flexibility, stretchability, durability and sensitivity, showing great prospects for soft sensors to be applied in robots [10,11], biomedical devices [12,13], and wearable devices [14]. The latest development of soft sensors, such as torsional or twisted sensors, is very promising, especially in real application. For instance, Cooper et al. [15] developed stretchable capacitive sensors to be used with artificial muscles and soft robotics. Do et al. [16] demonstrated stretchable electronic devices which are capable of sensing contact forces and distributed contact areas when devices were woven into textile. Atalay et al. [17] fabricated a textile-based soft sensor to monitoring human joint movement during the different scenarios. Zhou et al. [18] developed a liquid metal (LM)-based flexible sensor using printing technology, which had good practical application prospects in pose detection. The tactile sensor, as a major class of soft sensor, allows itself to be naturally applied to the human–robot interfacing. According to the sensing mechanisms, the flexible tactile sensor can be classified as piezoresistive [19,20], capacitive [21], and piezoelectric sensor [20], etc. Most piezoelectric and capacitive sensors can obtain better linear response, faster dynamic response, and high spatial resolution [22]. However, these sensors require a complex fabrication process and sophisticated circuit design [21]. On the contrary, owing to the well-established manufacturing and the relatively mature carbon-based nanomaterials, the functionalized piezoresistive sensor is able to offer a wide pressure-sensing range during a small deformation [23,24]. In addition, the simplified readout electronics has been investigated to some extent, leading to the extensive use of piezoresistive transduction mechanism in most of the commercially available tactile sensors [25]. However, hysteresis phenomena and crosstalk among the sensor elements result in insufficient sensitivity and repeatability, which limit their implementation in advanced human–robot interaction [26,27].

**Figure 1:**
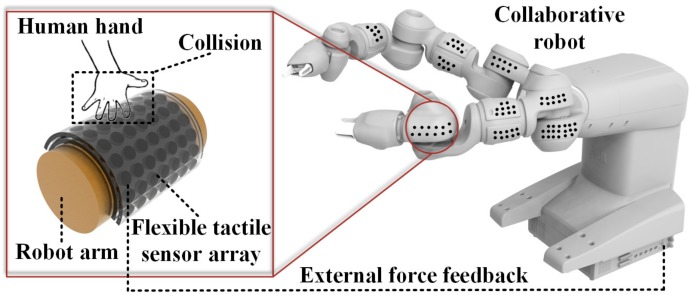
The flexible tactile sensor to endow YuMi robots with the perception of external force [8].

In this article, we propose a strategy for safe and natural human–robot interaction where a YuMi robot is endowed with a tactile human skin, as shown in Figure 1. To achieve this strategy, we developed a robot skin (tactile sensor array) to realize the external force perception. The robot skin was heterogeneously integrated with flexible conductive interconnections using inkjet printing. The soft substrate made by polydimethylsiloxane (PDMS) was able to achieve the conformal contact with collaborative robot. To reduce the potential risks of the impairments on the human body, we designed a compliant structure, which in this case is three-dimensional (3D) dome structure. The dome structure enhances the sensitivity of the robot skin to significant contact force variations. Soft materials and compliance structure have the effect of buffering energy during a collision, which can reduce the damage to the human body. This article aims to achieve multi-level security by integrating flexible sensor array with YuMi robot which has been embedded with torque sensors already. The developed 3D flexible sensor array could be integrated into the essential parts of YuMi robot, especially for the high-risk areas of collision.

The rest of the article is organized as follows. The main design features, working principle, and fabrication process are described in Section 2. The robot skin’s characteristics and collision testing are presented and discussed in Section 3. Finally, future work and conclusion are introduced in Section 4 and Section 5.

## 2. Materials and Methods

### 2.1. Structure Design and Working Principle

In this work, a dome structure is adopted as the basic 3D structure of the sensing unit. Aside from a dome structure, a micropillar structure is also a promising approach. However, this approach requires a silicon mold fabricated by conventional photolithography and etching, which is a time-consuming and complex process [28]. The typical form of micropillar sensor is film-like or cube-like structure, which limits their characteristics of compliance and compressing softness and applications in monitoring the collision impairments [29]. Additionally, most micropillar sensors were aimed at detecting subtle signal with high sensitivity, which does not correspond to the requirements of collision detection [30]. Compared to the micropillar approach (Table S1), the dome structure has the advantage of a simplified fabrication process, sufficient sensitivity to medium force (>3 N), and enhanced structural compliance to buffer energy.

**Figure 2:**
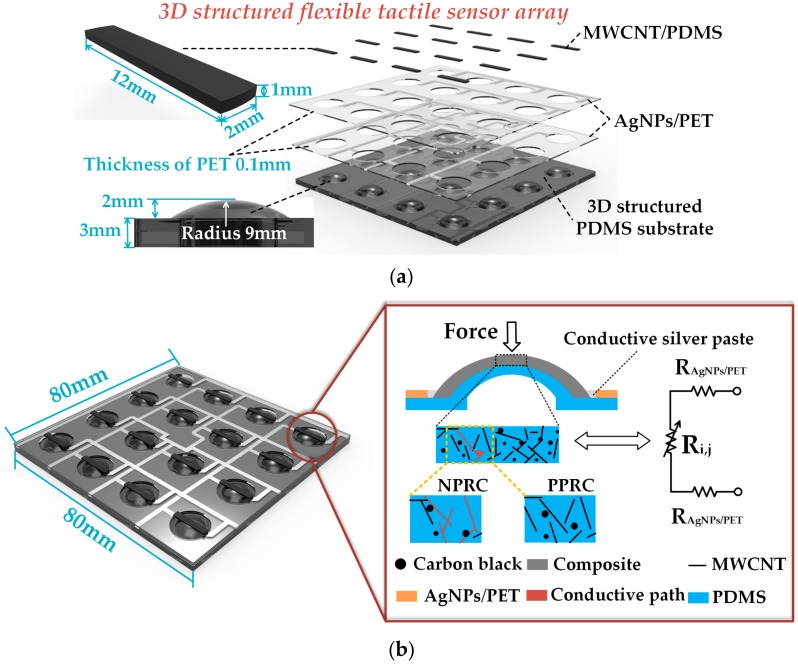
A schematic view of the flexible tactile sensor array: (**a**) The design of 3D structural flexible tactile sensor array; (**b**) The working principle of the 3D structural flexible tactile sensor unit.

The structure of the flexible tactile sensor array is demonstrated in Figure 2a. The hierarchical structure of the sensor array consists of the 3D structural PDMS substrate, the printed silver nanoparticles (AgNPs) traces on polyethylene terephthalate (PET) for the conductive interconnections (AgNPs/PET) of the sensing elements, the force sensitive nanocomposite layer prepared by embedding multi-walled carbon nanotubes (MWCNTs), and a spot of carbon black (CB) into PDMS (MWCNTs/PDMS). The two layers of AgNPs/PET are orthogonal to each other so that the row-column nodes are regarded as 16 pressure sensing islands. The total length and width of the sensor array are both 800 mm. The radius of the domed structure on PDMS substrate is 9 mm. The total thickness of the structured PDMS substrate is 5 mm. Each AgNPs/PET electrode has four conductive traces with a line width of 1.5 mm, and a total thickness of 0.1 mm.

More details of the working principle are shown in Figure 2b. When loaded with external force, the resistance of the sensitive composite changes in the process of deformation of the sensing unit. To reduce the risk of injury, the collaborative robot should perceive a collision in time. In other words, the proposed robot skin should have the ability to detect significant contact force variation. Compared with the structure of solid spherical crowns, large deformations are more likely to occur at the same load with the dome structure, resulting in a more significant change in the resistance of the sensitive nanocomposite (Figure S1). There are two trends in the resistance of the composite depending on the selected materials, the designed structure and the utilized method of manufacturing. One of the trends is a positive pressure resistance coefficient (PPRC) change, which means that the conductive paths formed by cross-linked MWCNTs and CB is decreasing under additive external force. Another trend is the negative pressure resistance coefficient (NPRC) change, which shows increasing conductivity as the load increases. Since the two trends depend on numerous factors, it is tough to explain the mechanisms without actual experiments [31]. The PPRC trend is demonstrated in this article. The electrical resistance of each MWCNTs/PDMS composite (R_i,j_) is 6–10 kΩ, whereas each AgNPs/PET conductive connector has a resistance (R_AgNPs/PET_) below 30 Ω, which means that the effect generated by AgNPs/PET connectors on the sensor is negligible.

### 2.2. Fabrication Process

According to the application requirements in this article, the Young’ modulus of PDMS can be adjusted by changing the ratio of pre-polymer and the cross-linker, which is the most important reason for choosing PDMS as the substrate material. The flexible PDMS substrate was fabricated by molding. As the thickness of the PDMS film is 3 mm, using a metal mold is likely to cause cracking of the film. Therefore, the mold used in this paper is obtained by stereolithography 3D printing technology based on photocurable resin. Compared with a metal mold, the photocurable resin mold has less adhesion force to PDMS, which means that PDMS can be remolded easily. In addition, stereolithography 3D printing is much cheaper than traditional metal machining. Besides, the surface quality of the resin mold fabricated by the stereolithography method is higher than that of the fused deposit 3D printing. It should be noted that a releasing agent should be sprayed on the surface of the mold. The mold should be designed with an appropriate number of pores to allow bubbles to be released from PDMS during curing. This will prevent bubbles forming in the PDMS substrate. The specific fabrication process mainly consists of the following steps:

Step 1: PDMS (Sylgard 184, Wuxi Changxu Technology Co., Ltd., Wuxi, China) was used with the weight ratio of pre-polymer and the cross-linker as 10:1, which was mechanically stirred for 5 min.

Step 2: The mixture of the pre-polymer and cross-linker was ultrasonically shaken and dispersed in an ultrasonic disperser (Ningbo Xinzhi Biotechnology Co., Ltd., Ningbo, China).

Step 3: The dispersed mixture was poured into the lower mold and degassed in a vacuum oven (Bangxi Instruments Technology Co., Ltd., Shanghai, China) for 10 to 15 min.

Step 4: The upper mold was covered over the lower mold. Following that, we applied a constant force (≈20 N) generated by 2 kg weight for 30 s to overflow the excess PDMS.

Step 5: A weight (1 kg) was placed on the surface of the upper mold to strengthen the fit between the mold and PDMS. The assembled molds with PDMS were placed in a vacuum drying oven and curing at 70 °C for 5 h.

Step 6: Finally, we took out the molds from the oven, separated the upper mold and lower mold, and carefully peeled off the flexible PDMS substrate with tweezers.

The flexible conductive trace was patterned using an inkjet printer (Functional nanomaterial deposition system, Shanghai Ruidu Photoelectric Technology Co., Ltd., Shanghai, China) to print AgNPs ink (PrintPlus–Ink10, Nanjing JiCang Technology Co., Ltd., Nanjing, China) on PET at 135 °C. The line width was 1.5 mm. At the corner of the conductive trace, the angle was 45 degrees to reduce the electromagnetic interference of the signal (Figure S2). After printing repeatedly for 5 times, we measured the resistance value of the conductive trace by a multimeter (Keysight Truevolt 34461A, Keysight Technologies, Shanghai, China). It was found that the resistance between the endpoints of each electrical connection for the sensing elements in the AgNPs/PET was less than 30 Ω. After being packaged with double-sided tape, the two AgNPs/PET conductive interconnections were cut to fit the PDMS substrate using laser cutting (FD–300, Shanghai Fengying Computer Technology Development Co., Ltd., Shanghai, China). The printed AgNPs/PET conductive traces on PET exhibited good stability (no severe resistance fluctuations and open circuits) and conductivity (<10 Ω) even after complete folding and pressing (<9 N), as shown in Figure S3.

To fabricate the force sensitive nanocomposite, we embedded 4 wt% MWCNTs (MK1858, Nanjing Muke Nano Technology Co., Ltd., Nanjing, China) and 1 wt% CB (Black pearls 2000, Cabot China Ltd., Shanghai, China) into PDMS. Firstly, the mixture of MWCNTs and CB as well as PDMS was dispersed with the ultrasonic disperser in a cold-water bath which absorbs the heat generated from ultrasonic treatment and to prevent PDMS curing during dispersal. The air bubbles in the mixture were evacuated in a vacuum oven for 10 to 15 min. Secondly, a uniform nanocomposite film with a thickness of 1 mm was prepared using a coating machine (AT–TB–1, Shandong Anne Mate Instrument Co., Ltd., Shandong, China) with optimized manufacture parameters (such as the speed of coating and the thickness of film) discussed in Figure S4. Following that, the nanocomposite film was placed in a vacuum oven and cured at 65 °C for 1 h. Finally, the sensitive unit was cut from the nanocomposite film with scissors and the size of it was 2 mm × 12 mm. Most importantly, if the contact resistance on the surface of the fabricated nanocomposite is unstable, which is caused by nonuniform dispersion of filler in the matrix near the surface, it can be surface-modified with a laser engraving machine to remove the nonuniform surface. This is a very effective way to obtain stable nanocomposite film.

**Figure 3:**
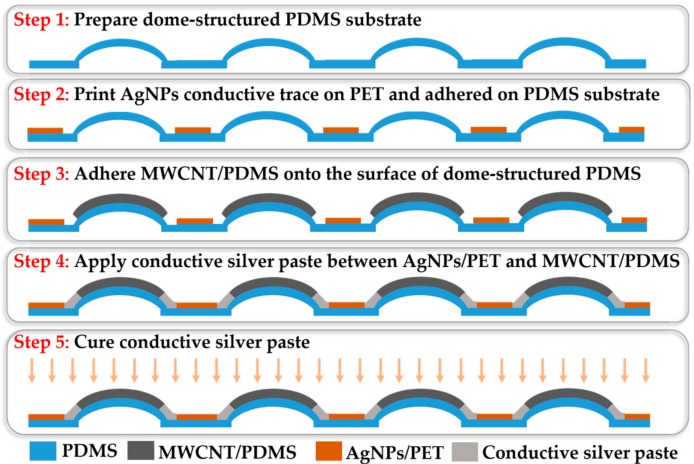
The integration steps of the flexible tactile sensor array.

**Figure 4:**
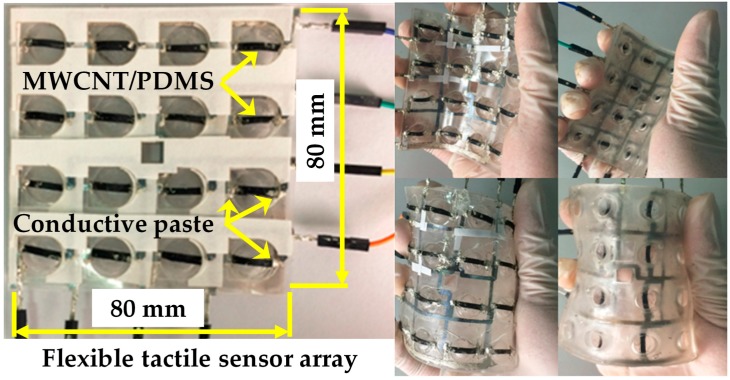
Photograph of the integrated flexible tactile sensor array.

After the fabrication of the flexible dome structured substrate, the two AgNPs/PET conductive interconnections, and the force sensitive elements, the flexible tactile sensing array was assembled, as shown in Figure 3. Firstly, the two layers of flexible AgNPs/PET conductive interconnections and dome-structured PDMS substrate were assembled. Two AgNPs/PET conductive interconnections were directly bonded with double-sided tape. Then, the bonded AgNPs/PET conductive interconnection was attached to the PDMS substrate using a silicon adhesive (Sil–Poxy, Smooth-on, Inc., Macungie, PA, USA). After that, the sensitive elements were adhered to the surface of the flexible PDMS substrate using the silicone adhesive curing at room temperature for 30 min. Then, conductive silver paste (Nanhai ETEB Technology Co., Ltd., Foshan, China) was glued between the endpoints of the AgNPs/PET conductive interconnections and the sensitive elements. Finally, the assembled device was placed in a vacuum drying oven at 80 °C for 15 min to cure the conductive silver paste. The integrated piezoresistive tactile sensor array exhibited good flexibility, enabling it to be attached to the arm of YuMi robot, as shown in Figure 4.

### 2.3. Tactile Sensing System

**Figure 5:**
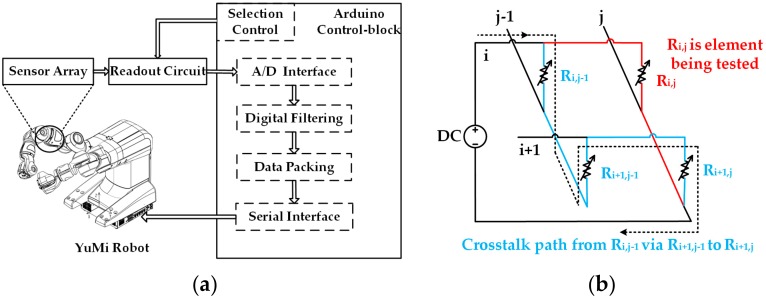
The schematic of the tactile sensing system: (**a**) Real-time sensing system for YuMi Robot; (**b**) A schematic of bypass crosstalk.

Figure 5a exhibits the schematic of the real-time flexible tactile sensing system to test the developed sensor array. The raw data of sensing element being tested from the readout circuit is packaged and transferred by the Arduino through the integrated analog-to-digital converter (ADC). The digital voltage signals are sent to the computer (or YuMi Robot) through the serial interface. The further signal processing, storage, and display in a real-time manner is accomplished by computer software. Generally, bypass crosstalk is an essential issue in the measurement of the distributed resistive sensor array loaded with the direct current (DC). There are two or more conductive loops during the measurement. In addition to the target element being tested (R_i,j_), the non-target resistors also constitute one or more loops (from R_i,j−1_ via R_i+1,j−1_ to R_i+1,j_), inducing the measurement error interference, as shown in Figure 5b. One solution to address this issue is to construct an equipotential shielding circuit to suppress bypass crosstalk through output voltage feedback applied on the non-selected lines [32].

**Figure 6:**
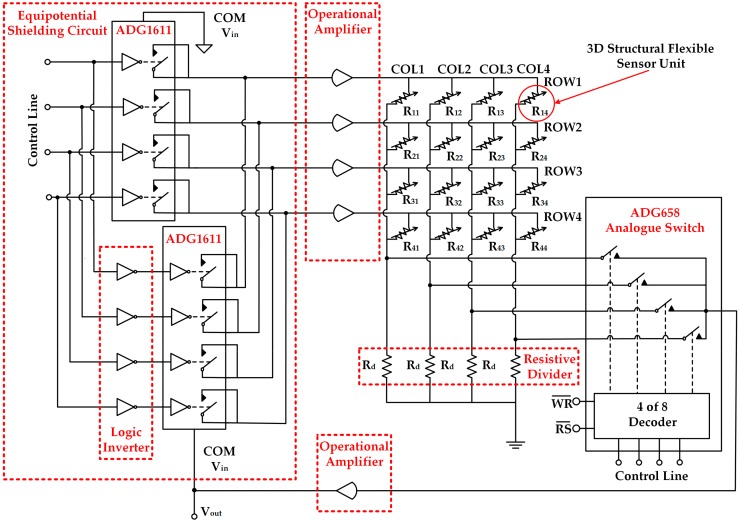
The schematic of the readout circuit schematic for the 4 × 4 3D structural flexible tactile sensor array. The controlled ADG1611 in the equipotential shielding circuit is adopted to select the row of the target resistance unit. The ADG658 selects the column of the target resistance. The resistance value of each resistive divider (R_d_) is set up to 10 kΩ, which is slightly greater than or equal to the resistance value of the target sensing unit. The amplification ratio of each amplifier is set up to 1.

**Figure 7:**
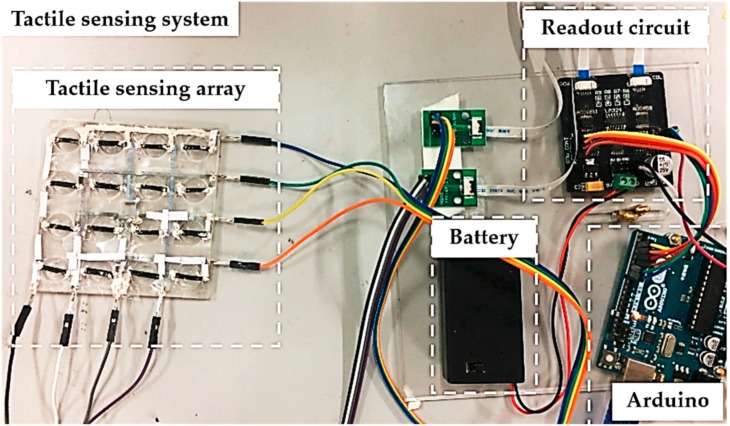
Photograph of the hardware configuration for the flexible tactile sensing system.

More details of the readout circuit are shown in Figure 6. The designed readout circuit was based on equipotential shielding method, including two four-channel analog switches (ADG1611, Analog Devices, Inc., Norwood, MA, USA), two operational amplifiers (LM324, Texas Instruments, Dallas, TX, USA), an eight-channel analog switch (ADG658, Analog Devices, Inc.), and a six-channel inverter (74LS04, Texas Instruments). The element being tested of the sensor array is selected by the ADG1611 and ADG658 with the scanning control signal generated by Arduino, and the voltage feedback reveals the resistance variation of the element being tested under external force load. The equipotential shielding circuit consists of two four-channel analog switches and an inverter. The row (ROW) and column (COL) of the target resistance unit is selected by controlling the analog switch (ADG1611 for row selection and ADG658 for column selection). The logic inverter makes the potential of the elements on all other rows equal to the output voltage of ADG658. The potentials on all other rows are clamped at the output voltage, which means that there is no deviation of potential among the other rows so that the elements on the other rows do not affect the current flowing through the target resistance unit. Finally, the true value of the target resistance unit R_i,j_ can be obtained from the ADG658 output. The resistance of the element being tested (R_i,j_) can be calculated as:

R_i,j_ = (V_in_/V_out_ − 1) × R (1)

where R is the voltage divider, V_in_ is the ADG1611 COM terminal load voltage (5 V), and V_out_ is the amplified ADG658 output voltage. The divider resistor should be chosen to be slightly greater than or equal to the resistance value of the target sensing unit, otherwise the measurement result will have a great error. The hardware configuration of the flexible tactile sensor array is shown in Figure 7.

## 3. Results and Discussion

### 3.1. Tactile Sensor Unit Calibration

**Figure 8:**
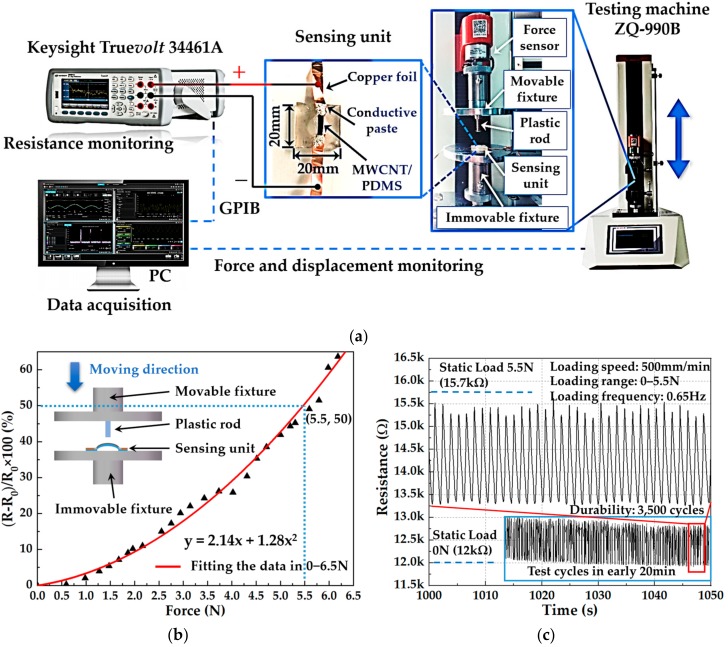
The calibration and stability test of tactile sensor unit: (**a**) The configuration of the testing system; (**b**) The calibration of the tactile sensor unit; (**c**) The stability and reproducibility test of the tactile sensor unit.

Since each sensing unit in the piezoresistive flexible tactile sensor array has the same structure and manufacturing process, this article conducted calibration on the sensing unit. The configuration of the test platform is mainly composed of a tension and compression testing machine (ZQ-990B, Zhiqu Precision Instrument Co., Ltd., Dongguan, China), a digital multimeter, and a computer (PC), as shown in Figure 8a. The sensing unit was fixed on the surface of the immovable fixture integrated on the testing machine and connected with the digital multimeter through copper foils and wires to monitor the change of resistance during the loading-unloading test. The surfaces of the fixture integrated on the testing machine was insulated by pasting PET tape to prevent the movable and immovable fixtures made by conductive metal materials from affecting the resistance measurement. At the same time, a 3D printed plastic rod was fixed on the surface of the movable fixture, which was used to load force on the sensitive layer on the surface of the sensing unit. The plastic rod mounted on the testing machine moves in the vertical direction with the movable fixture. When the movable fixture moves downward, the plastic rod is brought into contact with the sensitive layer of the sensing unit, thereby generating a changeable contact force. Furthermore, the contact force was recorded by the force sensor integrated into the testing machine. All raw data of the contact force and resistance were sent to the PC software for further data processing, storage, and analysis. Then, the calibration of the tactile sensor unit was carried out.

The initial resistance value (R_0_) of the sensing unit was 6.5 kΩ before loading. After sampling the resistance value (R) during one-step loading, the test results are shown in Figure 8b. The graph shows the one-to-one correspondence between resistance output and force input in the range of 0–6.5 N with the maximum force sensitivity of 18.83% N^−1^ that was calculated from the derivative of the fitted function at 6.5 N shown in Figure 8b. It should be noticed that the sensitivity or gauge factor (GF) of our sensor is defined as percentage change in impedance per Newton force (GF = (∆R/R_0_)/∆F, where ∆R/R_0_ is the percentage change in impedance and ∆F is the applied force variation). Testing of reproducibility and stability was conducted with the same testing platform as calibration, whereas the test sample was different. The initial resistance of the sensing unit used in stability test was 12 kΩ. When being loaded with an external force of 5.5 N, the sensing unit showed an increased resistance of 15.7 kΩ. During the experiment, the loading range was 0–5.5 N and the loading speed was 500 mm/min. The test results of stability and reproducibility at the beginning are shown in Figure 8c. The developed sensing unit showed a sufficient durability of 3500 cycles. The response curve of sensing unit after 3500 cycles is demonstrated in Figure S5b. During the repetitive loading process, the curve of resistance of the sensing unit showed a good periodicity. The frequency resistance signal was calculated as 0.65 Hz. However, the resistance value of the sensitive layer varied from 12 kΩ to 15.7 kΩ. When the dynamic load force became 0 N, the resistance of the sensitive layer was greater than the resistance corresponding to the static load of 0 N. When the dynamic load force became 5.5 N, the resistance of the sensitive layer was less than the resistance corresponding to the static load of 5.5 N. This phenomenon was caused by the viscoelasticity of the substrate material. During the multiple loading-unloading cycles, the testing machine applied force to the sensing unit by driving the movable fixture up and down, whereas the deformation speed of the substrate material was relatively slow due to the viscoelasticity of the PDMS, resulting in a small range of resistance change. The low-frequency stability tests are demonstrated in Figure S5a. Finally, an extra durability test was conducted with the same platform, of which the loading range was set as 0–2.5 N under a frequency of 1 Hz. The developed sensing unit showed good durability without losing sensing function after 10,000 cycles of the loading-unloading test as shown in Figure S5c,d.

### 3.2. Tactile Sensor Array Response

**Figure 9:**
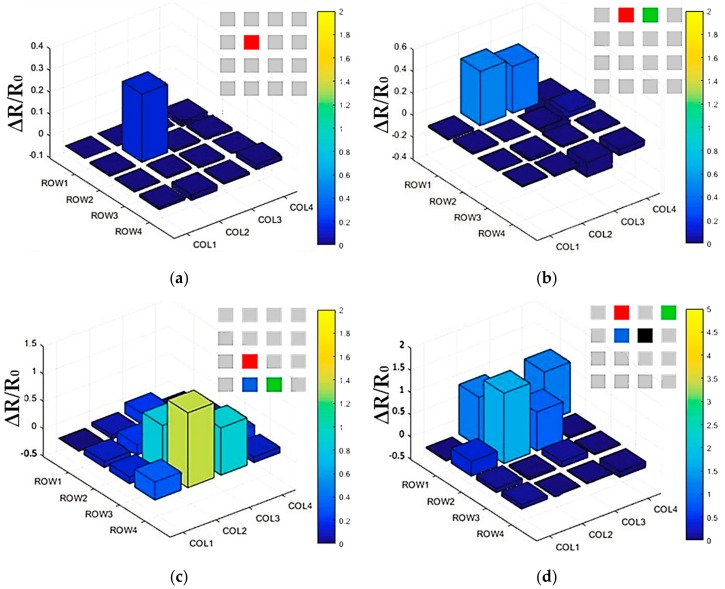
The distribution of tactile data from the flexible tactile sensor array during finger touching under different conditions: (**a**) Only one sensing unit being tested; (**b**) Two sensing units being tested; (**c**) Three sensing units being tested; (**d**) Four sensing units being tested. Illustration in each picture shows the finger touch positions.

The finger touch test was carried out to verify the function of the developed flexible tactile sensing system, including contact force sensation of the flexible tactile sensor array and the real-time data acquisition and visualization, as shown in Figure 9. The vertical axis in each picture indicates the change in the resistance value (ΔR) of each sensing unit in the form of a percentage. The resistance value of the corresponding sensing unit increased obviously when the sensing unit was pressed at different positions, which was clearly shown in the 3D histogram. The color of the 3D histogram in each chart is to better represent different values of contact force of each sensing unit. Due to the flexibility and stretchability of the tactile sensor array, the whole system introduced small errors during the finger touch test, which was reflected in the small fluctuation of the other sensing units that were not pressed. Compared with the percentage change of target sensing unit, these errors (ΔR/R_0_ ≈ 0.3 at the node between the fourth row and the first column in Figure 9c) were negligible for the contact force detection of the target sensing unit (ΔR/R_0_ ≈ 1.4 at the node between the fourth row and the second column in Figure 9c). From the finger touch experiment, we can see that the whole flexible tactile sensing system could work normally, which supported the follow-up integration test of flexible robot skin for safe and natural human–robot collaboration.

### 3.3. Flexible Robot Skin Integration

**Figure 10:**
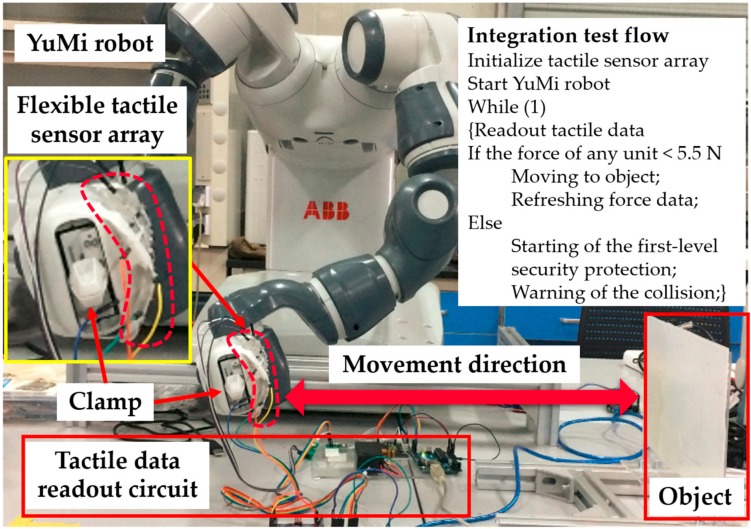
The configuration of the security test based on a YuMi robot integrated with the developed 3D flexible tactile sensor array.

According to the recommendations of the ISO/TS 15066 Collaborative Robot Technical Specification, the level of human–robot interaction can be improved by incorporating multiple sensing functions and constructing multi-level security assessment mechanisms. For example, on the basis of multi-sensing, such as the sensation of distance and contact force between the robot and human as well as the detection of the torque in each joint of robot, the collaborative robot can switch various working patterns. These patterns are full-speed and independent of human assistance, versus the slow-moving operation when someone enters the workspace of robot, an intuitive human–robot interaction when the mission requires the cooperation of humans and robots, and halting once collision occurs. Among these patterns, halting once collision occurs was further demonstrated by applying the developed robot skin to a YuMi robot to test security performance. The configuration of the security test consisted of the YuMi robot, the developed robot skin, the tactile data readout circuit, an object, and a PC for data storage, as demonstrated in Figure 10. The flexible robot skin was attached to the surface of the robot’s clamp. The object was a vertically placed flatbed coated with a layer of silicone rubber to mimic human skin. The clamp moved in the horizontal direction towards the object. During the integration test, the clamp moved towards the object and stopped when the collision occurred, the process of which was monitored by the flexible robot skin through the analysis of tactile data.

**Figure 11:**
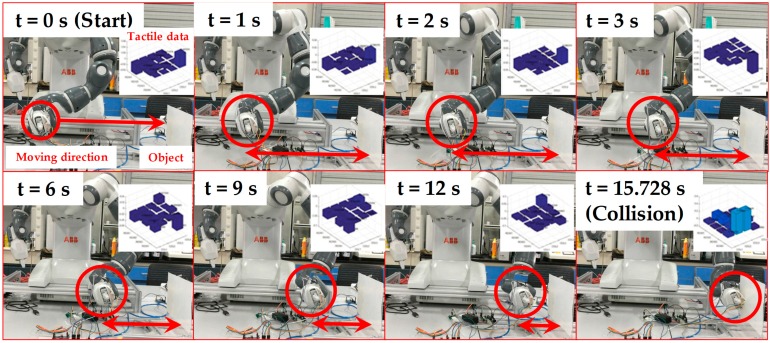
The integration test for safe and natural human–robot collaboration.

**Figure 12:**
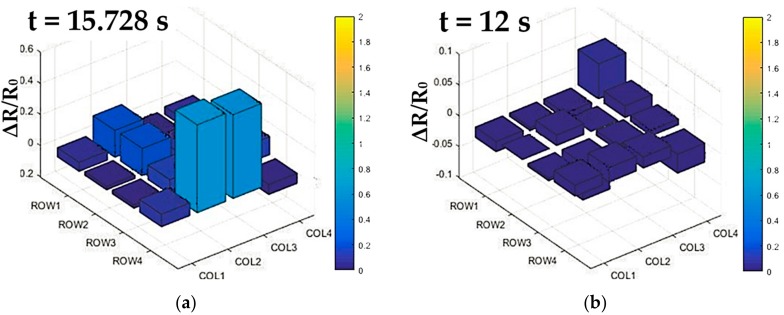
The tactile data from the 3D flexible tactile sensor array in a real-time manner during the security test: (**a**) Tactile data before the collision; (**b**) Tactile data when the collision occurred.

Due to the seamless integration of the flexible tactile sensor array and the clamp, the tactile sensor array was deformed with the curved surface of the clamp of YuMi robot, resulting in the baseline drift of tactile data before the test. Therefore, the flexible tactile array was firstly scanned once to obtain the initial resistance values of each sensing elements caused by deformation. Then, the measurements of tactile data were obtained by subtracting the initial resistance values from the current readings. Whether the clamp collided with the object was judged according to the percentage change of resistance of the flexible tactile sensor array. The judgment of this value was based on whether the force sensed from any sensing unit reached up to 5.5 N. According to the calibration of the sensing unit in Figure 8b, when the contact force was 5.5 N, the resistance change rate was 50%. If there was a sensing unit whose resistance change rate exceeded 50%, the YuMi robot would identify the collision and stop moving. The result of the integration test for safe and natural human–robot collaboration is shown in Figure 11. According to the experimental results of the integration test, some small errors (ΔR/R_0_ < 0.1 at the node between the first row and the fourth column in Figure 12a) were inevitably introduced in the integration test. These errors were generated by the small deformations that were caused by the motion of the robot and the connections used for the tactile data transmission. Fortunately, these errors did not affect the implementation of the security interaction strategy designed in this article. In this experiment, the YuMi robot realized the emergency stop successfully when the collision occurred. As showcased in Figure 12b, the resistance change rate was over 50% (56.5% at the node between the fourth row and the second column, 54% at the node between the fourth row and the third column), when the YuMi robot stopped moving.

## 4. Future Work

There are still some aspects that require further research to achieve intrinsically safe collaborative robots by investigating advanced multi-level security. Firstly, the structural design of the piezoresistive flexible tactile sensing array needs to be optimized to enhance the sensing performance. In terms of pixels, the distribution of sensing elements and conductive interconnections can be optimized and compacted by on-demand printing. In this article, the PDMS substrate has a thickness of 3 mm to prevent damage due to strong adhesive forces caused by large area contact between the PDMS and the molds during peeling. To optimize the thickness of substrate, the structural design of molds can be adjusted to reduce the contact area. Furthermore, the dome-structured substrate will be improved by designing and fabricating a high-spatial-resolution mold. To expand real application in advanced human–robot interaction, an enhancement method to obtain high sensitivity through changing the substrate materials/stiffness and radius of dome structure should be explored. Additionally, multi-sensing heterogeneous integration, such as an ultrasonic sensor for distance sensation, is required to establish the advanced multi-level security assessment mechanisms. Besides, the wireless connection between the flexible sensing system and the PC/robot used for the corresponding data processing should be developed to avoid interfering with the workspace of the collaborative robot. Lastly, extensive coverage of the flexible tactile sensor array on the surface of the robot needs to be implemented to improve safe and natural human–robot collaboration.

## 5. Conclusions

For safe human–robot collaboration, this article designed a security strategy based on a flexible tactile sensor to enhance the perception of external force by collaborative robots. We proposed a dome structure to enhance the sensitivity and compliance of a piezoresistive flexible robot skin. In addition, the manufacturing process of the dome-structured tactile sensing array was based on inkjet printing and molding methods, including heterogeneous integration of the components with different materials. We also designed the readout circuit to suppress bypass crosstalk according to the equipotential shielding method. The hardware implementation of the flexible tactile sensing system for contact force visualization was achieved. Furthermore, the sensing performance of the developed tactile sensor was tested, which exhibited a one-to-one relationship between force input and resistance output in the range of 0–6.5 N with a maximum force sensitivity of 18.83% N^−1^ at 6.5 N. The fabricated sensing unit showed good reproducibility after loaded with cyclic force (0–5.5 N) under a frequency of 0.65 Hz for 3500 cycles. Finally, a collision test was conducted with YuMi robot, which verified that the flexible tactile sensing system could achieve halting once collision occurred. All the experimental results showed that the developed flexible robot skin could offer an efficient way for natural and safe human–robot interaction.

## Supplementary Materials

The following are available online at http://www.mdpi.com/2072-666X/9/11/576/s1, Figure S1: Finite element method (FEM) simulation for stress-strain analysis, Figure S2: Photograph of the two printed AgNPs/PET conductive interconnections, Figure S3: Conductive traces stability test, Figure S4: Piezoresistive nanocomposite film uniformity test, Figure S5: The stability and reproducibility test of tactile sensor unit, Table S1: Summary of typical soft sensors based on micropillar approach, Video S1: Demonstration of integration test with YuMi robot.
