# Supplementary material for: Development of Flexible Robot Skin for Safe and Natural Human–Robot Collaboration
Source: Micromachines (Basel). 2018 Nov 5;9(11):576. doi: 10.3390/mi9110576 (PMC6266199; doi:10.3390/mi9110576)
Supplement: Supplementary file 1 [file micromachines-09-00576-s001.zip › micromachines-377280-SI/Micromachines-377280 Supplementary Materials.pdf]

Supplementary Materials: Development of Flexible Robot Skin for Safe and Natural Human-robot Collaboration

Gaoyang Pang, Jia Deng, Fangjinhua Wang, Junhui Zhang, Zhibo Pang and Geng Yang

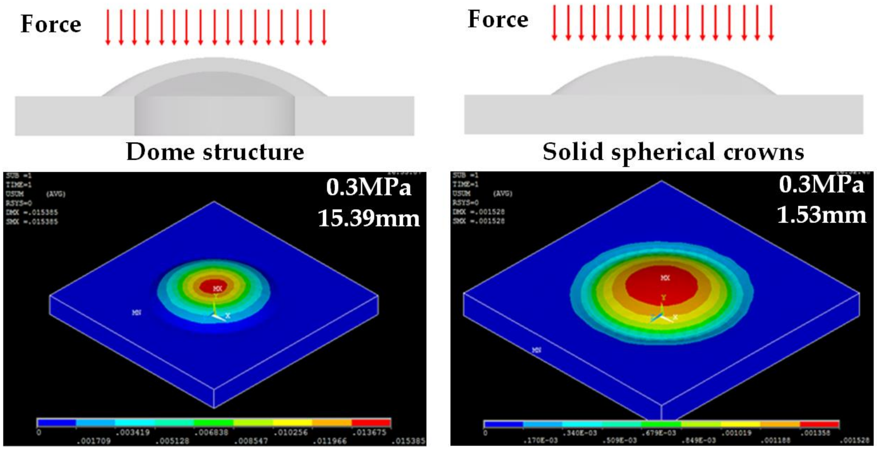

Figure S1. Finite element method (FEM) simulation for stress-strain analysis. The FEM simulation processes of three-dimensional (3D) dome structure and solid spherical crowns were conducted by ANSYS (Ansys, Inc., Cecil Township, PA, USA). The analysis results of FEM simulation show that the dome structure will produce larger deformation and stresses than the solid spherical structure after being applied the same load.

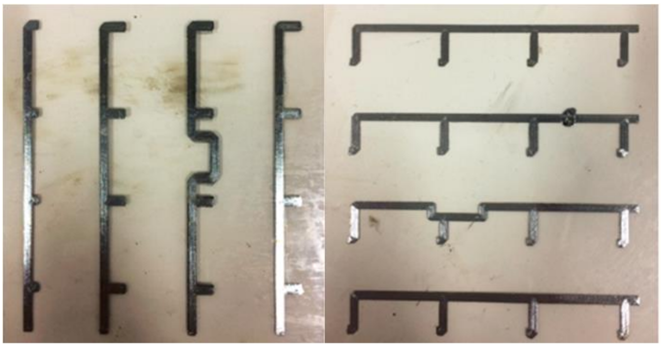

(**a**)

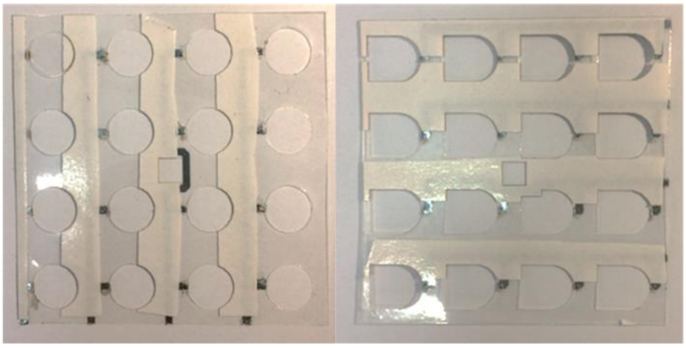

(**b**)

Figure S2. Photograph of the two printed conductive interconnections: (**a**) The conductive traces on flexible polyethylene terephthalate (PET) substrate after 5 times printing; (**b**) The packaged conductive interconnections by double-side paste.

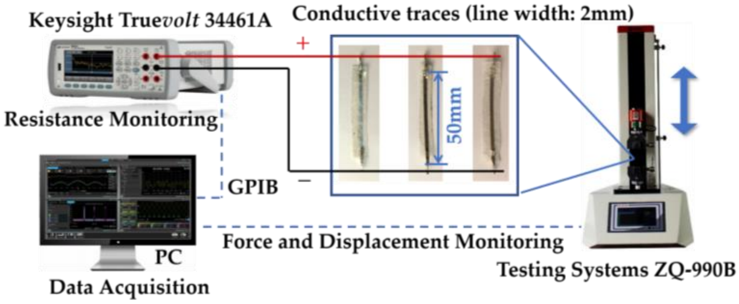

(**a**)

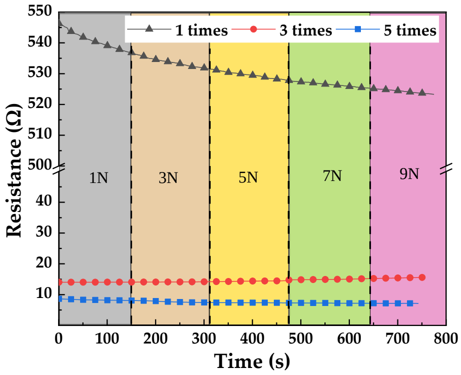

(**b**)

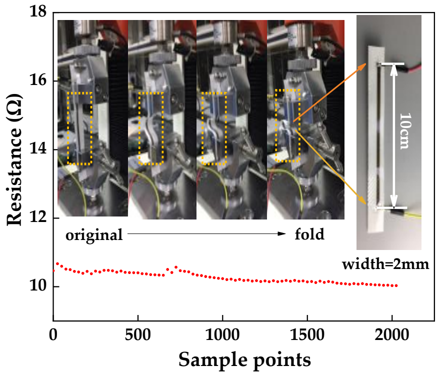

(**c**)

Figure S3. Conductive traces stability test: (**a**) Experimental scheme and method; (**b**) Compression test result showing that after 3 times printing the conductivity was stable under 20Ω during loading the compressive external force from 1N to 9N. The test samples had a length of 50mm with a line width of 2mm, which were formed by printing silver nanoparticles (AgNPs) ink on PET and packaging with a double-sided tape; (**c**) Bending test result showing that after 5 times printing the conductivity was stable under 10Ω during bending and folding. The test sample had a length of 10cm with a line width of 2mm, which was formed by printing AgNPs ink on PET and packaging with a double-sided tape.

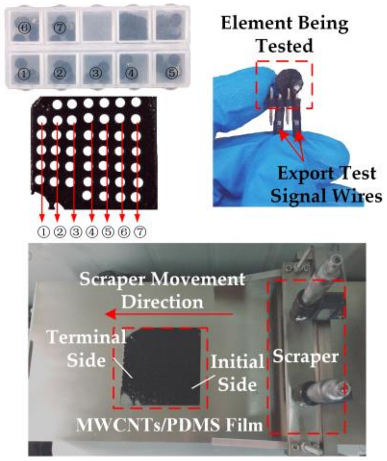

(**a**)

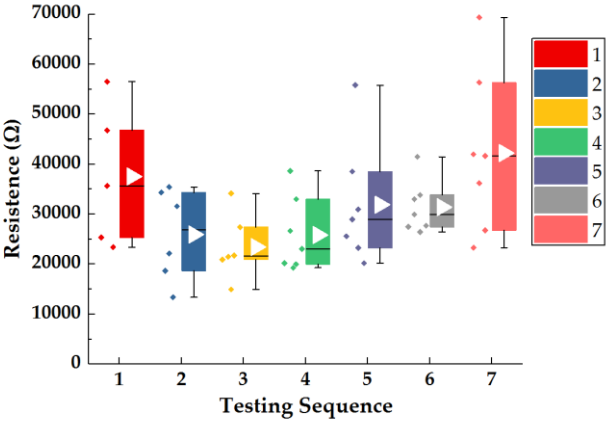

(**b**)

Figure S4. Piezoresistive nanocomposite film uniformity test: (**a**) Experimental method and procedure. We took several samples of the same geometric size along the direction of the scraper movement. Only one dimension, that is, scraper movement direction analysis, was discussed because the vertical effect is negligible; (**b**) The resistance value distribution of the piezoresistive nanocomposite film in the direction of the scraper, which shows that the resistance of both ends of the film is relatively large and the middle resistance distribution is more uniform.

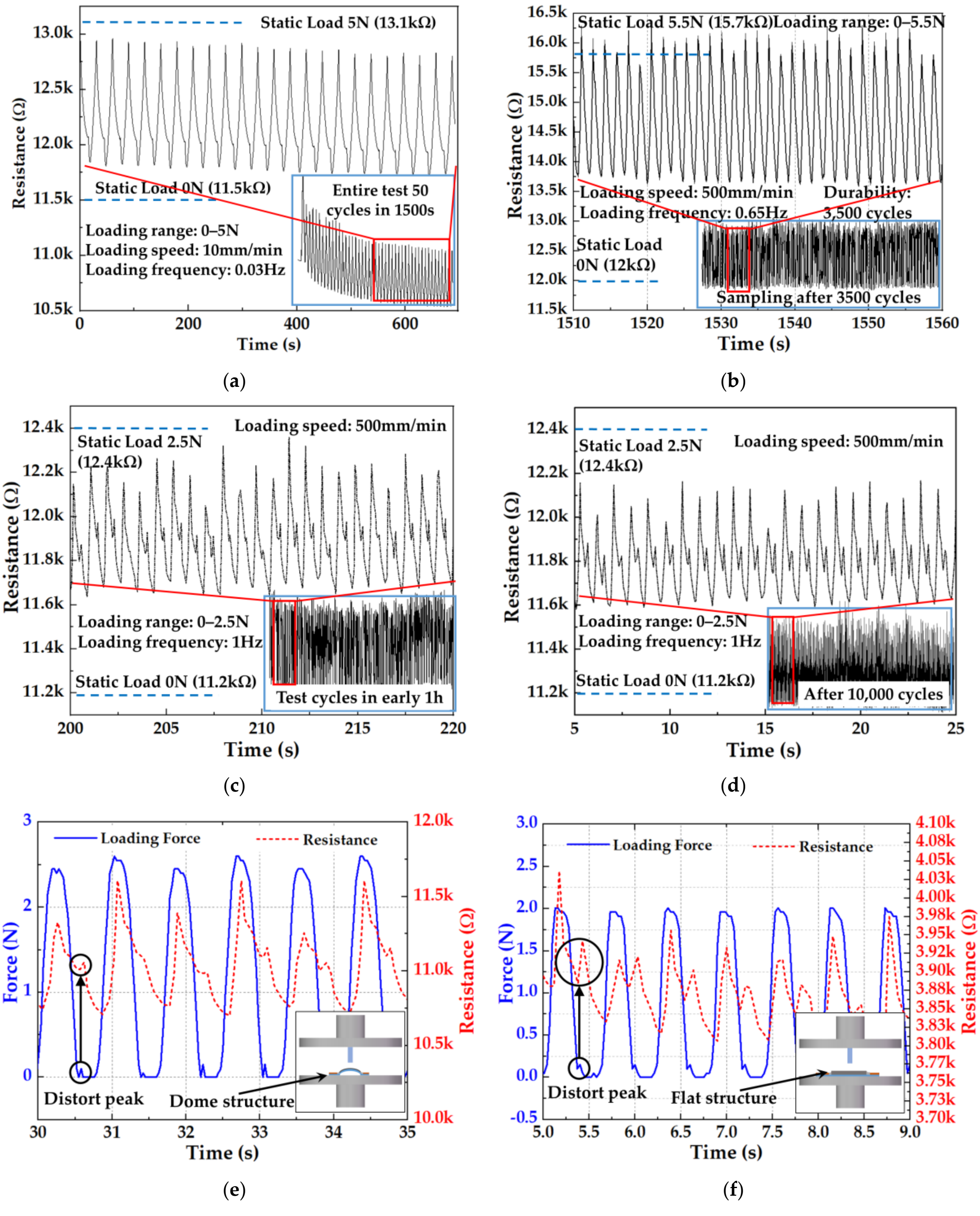

Figure S5. The stability and reproducibility test of tactile sensor unit: (**a**) Test results under a loading frequency of 0.03Hz; (**b**) Durability test results of 3,500 loading-unloading cycles under a frequency of 0.65Hz in a force range of 0–5.5N; (**c**)–(**d**) Durability test results of 10,000 loading-unloading cycles under a frequency of 1Hz in a force range of 0–2.5N; (**e**) Test results of dome structured sensor unit; (**f**) Test results of flat structured sensor unit. Notes: the sample tested in (**b**) is the same one in Figure 8c. The sample tested in (**c**) and (**d**) are same. It should be noticed that the results in (**c**) and (**d**) show distort peak with increase of frequency. To explain the distort peak, extra two tests have been conducted, as shown in (**e**) and (**f**). Test result in (**e**) where we applied a dome structured sensor unit shows that distort peak in resistance is unrelated to sensing materials because of corresponding distort in force. Test result in (**f**) where we applied a flat structured sensor unit shows that distort peak in resistance is unrelated to sensor structure. These two results show that the distort peak is caused by the intrinsic limitation (test frequency range is below 0.65Hz) of testing machine (ZQ–990B, Zhiqu Precision Instrument Co., Ltd., Dongguan, China) rather than the developed tactile sensor.

Table S1. Summary of typical soft sensors based on micropillar approach.

Approach	Manufacturing of mold	Sensing range	Feature	Application	Ref.
Micropillar	Photolithography and etching	0–10N	High sensitivity at 0–2N	Gentle touch sensor	[1]
Micropillar	Photolithography and etching	0–0.32N	High skin conformability	Pulse signal amplifier	[2]
Micropillar	Photolithography and etching	0–12N	One-pixel size of 37 mm × 37 mm	Human motion monitor	[3]
Micropillar	Photolithography and etching	0–0.45N	High sensitivity at low-pressure regions	Pressure-sensitive artificial skin	[4]
Micropillar	Photolithography and etching	0–6.4N	High sensitivity at low-pressure regions	Gentle touch and vibration sensor	[5]
Micropillar	Photolithography and etching	0–2.5μN	Miniaturized size of 10 mm × 6 mm	Droplet sliding detector	[6]
Micropillar	Photolithography and etching	0–225μN	High sensitivity at low-pressure regions	Wrist pulse collector	[7]
Micropillar	Photolithography and etching	0–1.95N	High sensitivity at low-pressure regions	Electronic skin for robots	[8]
3D dome structure	Stereolithography 3D printing	0–6.5N	Enough sensitivity at 5–6.5N; One-pixel size of Φ8mm	Collision monitor for safe human-robot collaboration	this work

References

1. Azkar Ul Hasan, S.; Jung, Y.; Kim, S.; Jung, C.L.; Oh, S.; Kim, J.; Lim, H. A sensitivity enhanced MWCNT/PDMS tactile sensor using micropillars and low energy Ar(+) ion beam treatment. *Sensors* **2016**, *16*, 93–103, DOI: 10.3390/s16010093.
2. Pang, C.; Koo, J.H.; Nguyen, A.; Caves, J.M.; Kim, M.G.; Chortos, A.; Kim, K.; Wang, P.J.; Tok, J.B.; Bao, Z. Highly skin-conformal microhairy sensor for pulse signal amplification. *Adv. Mater.* **2015**, *27*, 634–640, DOI: 10.1002/adma.201403807.
3. Dhakar, L.; Gudla, S.; Shan, X.; Wang, Z.; Tay, F.E.; Heng, C.H.; Lee, C. Large scale triboelectric nanogenerator and self-powered pressure sensor array using low cost roll-to-roll UV embossing. *Sci. Rep.* **2016**, *6*, 22253, DOI: 10.1038/srep22253.
4. Shao, Q.; Niu, Z.; Hirtz, M.; Jiang, L.; Liu, Y.; Wang, Z.; Chen, X. High-performance and tailorable pressure sensor based on ultrathin conductive polymer film. *Small* **2014**, *10*, 1466–1472, DOI: 10.1002/smll.201303601.
5. Park, J.; Lee, Y.; Lim, S.; Lee, Y.; Jung, Y.; Lim, H.; Ko, H. Ultrasensitive piezoresistive pressure sensors based on interlocked micropillar arrays. *BioNanoSci.* **2014**, *4*, 349–355, DOI: 10.1007/s12668-014-0151-8.
6. Thanh-Vinh, N.; Takahashi, H.; Matsumoto, K.; Shimoyama, I. Two-axis MEMS-based force sensor for measuring the interaction forces during the sliding of a droplet on a micropillar array. *Sens. Actuators A Phys.* **2015**, *231*, 35–43, DOI: 10.1016/j.sna.2014.09.015.
7. Park, H.; Jeong, Y.R.; Yun, J.; Hong, S.Y.; Jin, S.; Lee, S.J.; Zi, G.; Ha, J.S. Stretchable array of highly sensitive pressure sensors consisting of polyaniline nanofibers and Au-coated polydimethylsiloxane micropillars. *ACS Nano* **2015**, *9*, 9974–9985, DOI: 10.1021/acsnano.5b03510.
8. Ha, M.; Lim, S.; Park, J.; Um, D.-S.; Lee, Y.; Ko, H. Bioinspired interlocked and hierarchical design of ZnO nanowire arrays for static and dynamic pressure-sensitive electronic skins. *Adv. Funct. Mater.* **2015**, *25*, 2841–2849, DOI: 10.1002/adfm.201500453.

© 2018 by the authors. Submitted for possible open access publication under the terms and conditions of the Creative Commons Attribution (CC BY) license (http://creativecommons.org/licenses/by/4.0/).
